# Impact of de-ionized water on changes in porosity and permeability of shales mineralogy due to clay-swelling

**DOI:** 10.1038/s41598-021-99523-2

**Published:** 2021-10-08

**Authors:** Di Zhang, Jay N. Meegoda, Bruno M. Goncalves da Silva, Liming Hu

**Affiliations:** 1 Department of Civil & Environmental Engineering, New Jersey Institute of Technology, Newark, NJ USA; 2 State Key Laboratory of Hydro-Science and Engineering, Department of Hydraulic Engineering, Tsinghua University, Beijing, China

**Keywords:** Environmental sciences, Engineering

## Abstract

Hydraulic fracturing is widely applied for economical gas production from shale reservoirs. Still, the swelling of the clay micro/nano pores due to retained fluid from hydraulic fracturing causes a gradual reduction of gas production. Four different gas-bearing shale samples with different mineralogical characteristics were investigated to study the expected shale swelling and reduction in gas permeability due to hydraulic fracturing. To simulate shale softening, these shale samples were immersed in deionized (DI) water heated to 100 °C temperature and subjected to 8 MPa pressure in a laboratory reactor for 72 hours to simulate shale softening. The low-temperature nitrogen adsorption and density measurements were performed on the original and treated shale to determine the changes in micro and nano pore structure. The micro and nano pore structures changed, and the porosity decreased after shale treatment. The porosity decreased by 4% for clayey shale, while for well-cemented shale the porosity only decreased by 0.52%. The findings showed that the initial mineralogical composition of shale plays a significant role in the change of micro and nano pores and the pore structure alteration due to retained fluid from hydraulic fracturing. A pore network model is used to simulate the permeability of shale used in this study. To define pore structure properties, specific factors such as porosity, pore size, pore throat distribution, and coordination number were used. Furthermore, the anisotropy characteristics of shale were integrated into the model via a coordination number ratio. Finally, the change in permeability due to shale softening was determined and compared with untreated with the progress of shale softening. The simulation showed that the permeability of Longmaxi shale could decrease from 3.82E–16 m2 to 4.71E–17 m^2^ after treatment.

## Introduction

During the past decades, due to the development of multi-stage hydraulic fracturing and horizontal drilling technologies, worldwide unconventional shale gas production in low permeability shale reservoirs has substantially increased^1^. According to the U.S. Energy Information Administration (EIA), the shale gas production in the USA accounts for 50% of total natural gas production^2^. Hydraulic fracturing involves the injection of high-pressure fracturing fluid into the shale formation to produce a complex fracture network, facilitating the extraction of adsorbed or stored shale gas in micro and nano pores. Approximately 50% of the fracturing fluid is recovered after hydraulic fracturing and the balance is retained in the shale formation^3^. Hence, a significant volume of fracturing fluids remains in the reservoir. The interaction of trapped fracturing fluids with the shale formation cause a reduction in the permeability and hence the gas productivity of the shale formation^4^.

Upon exposure to the aqueous fracturing fluids at high temperatures and high pressures, the mechanical properties of shales such as elasticity, hardness, and strength usually deteriorate due to a phenomenon called “shale softening,” which occurs within the shale matrixes^5^. Generally, shale softening significantly impacts the design and operation of shale gas exploration and long-term gas production. This reactivity between water-based fracturing fluids and shale formations is due to the water-sensitive clay minerals and acid-sensitive carbonate minerals in shale^6,7^. When shale matrix encounters water-based drilling and fracturing fluids, shale swelling and softening occur. As a result, the flow of shale gas can be significantly reduced. In other words, the pore structure of the shale would change with the interaction of fracturing fluids. Hence, the petroleum industry will benefit tremendously if the sensitivity of shale to water-based fluids can be quantified and the changes in the permeability of each type of shale can be predicted before fracking.

Although the current hydraulic fracturing technology for shale gas production has been widely used, the geochemical reactions and physical changes due to the shale-hydraulic fracturing fluid reaction and the factors contributing to those reactions are not well understood. Several studies have shown that the shale swelling is due to the interaction of retained and fracturing fluid with clays in shale. Zhang et al.^8^ analyzed impact of slick-water on pore structure due to clay-swelling and carbonate-dissolution. Sun et al.^9^ studied the different fracking fluids and additives for porosity and pore structure alteration and showed how fracturing fluids affect the mineralogical compositions and physical porosities of shale. Xu et al.^10^ studied the mineralized water cross-linked with borax effect on the reservoir formations. For shale softening, acidic fracturing fluid (pH 2.1) was used by Houben et al.^11^. Also, Jew et al.^12^ studied the impact of acid fluid with pH ranging from 2.0 to 6.5 on carbonate and organics. Dieterich and Marcon^13,14^ studied the alteration of Marcellus shale by the carbonate dissolution due to acidic fracturing fluid. The acid and clay stabilizer reaction on mechanical damage in shale was studied by Wick et al.^15^.

With clay minerals, the shale wettability is an important factor when softening is investigated, and it may change over time due to the hydration/osmotic processes associated with such clays^16^. Using both the Young formulation for air/water/shale contact angle measurements and the direct oil/water/shale contact angle measurements, it was concluded that all shale rocks, regardless of composition, are water-wettable with contact angle equal or greater than 90°^17^.

Due to retained fluid from hydraulic fracturing, exchangeable cations between clay layers of shale formations are hydrated, increasing the gap between clay layers. Fink et al.^18^, Krishna Mohan et al.^19^, Norrish^20^, Zhang, and Low^21^ have shown that the two active pathways of clay swelling and hence potential porosity reduction are due to crystalline and osmotic swelling. Wilson et al.^22^ showed swelling of North Sea sandstone formation due to clay content in the mineralogy. Davy et al.^23^ showed that fractures could heal when in contact with fracturing fluids for poorly connected sedimentary rocks with low porosities ranging from 1 to 5%.

Clay swelling hinders gas flow and reduces the effective permeability of shale^24,25^. Simultaneously, hydration weakens the binding of mineral particles, thereby further decreasing fracture aperture and hence hydraulic conductivity. Although laboratory-scale water-clay interaction experiments provide valuable preliminary data on the physical and chemical interactions between shale and fracturing fluid, most of these absorption experiments were performed at low temperatures and pressures which are not representative of actual gas bearing shale formations^26,27^.

Based on the above discussion, the influence of fracking fluid on pore structure could be attributable to two phenomena: clay mineral expansion and carbonate mineral dissolution. Due to the interaction of fracking fluids with shale, minerals can be swollen and dissolving at the same time, and their strength varies with soaking duration, resulting in variations in specific surface areas and pore sizes^28–30^. However, only a few studies have investigated the change in shale permeability due to clay-water reaction since there are many chemicals in the fracking fluid. The DI water would penetrate the shale matrix and cause expedited separation of clay particles in shale. Therefore, with the extreme clay water reaction, the permeability change due to clay-water reaction and shale softening can be predicted using an anisotropic pore network model. The shale softening and the predictions of gas production due to shale softening will be critical to the selection of fracturing fluids and gas well planning designs.

The primary objective of this study is to investigate the impact of mineralogy on the change in micro and nano porosity of shale and the change in permeability due to hydraulic fracturing, particularly due to the interaction between the fracturing fluid and the shale. The clay-water reaction is a dynamic reaction, with several controlling factors leading to variable test outcomes. Hence several high-temperature and high-pressure shale immersion tests were performed to quantify the impact of water-based drilling fluid on the physical properties of gas-bearing formations. Then low-pressure nitrogen absorption–desorption (BET) experiments, porosity, and density tests were performed on four different shales before and after treatment to measure variations in pore sizes and porosity distribution. Then anisotropic pore network was used to predict the change in shale permeability by utilizing the pore structural information obtained from the above experiments.

## Materials and methods

### Sample preparation

The four types of shale, namely Hayneville, Longmaxi, Eagle Ford, and Opalinus, that are from different part of the world were used in this study. The basis for the above selection was the relatively different lithological characteristics and organic contents. Drying and cracking of shale samples would occur if they were in contact with the water vapor in the environment. Hence core samples were stored in moisture-free containers to provide a water-free and constant-pressure environment before use. The block shale samples were cut into 1 cm^3^ volume small cubic pieces using a low-speed precision cutter (model type–minitom manufactured by Struers). This machine can produce accurate 1cm^3^ cubic shale specimens. An industrial-grade diamond blade was used to slice the bedding plane to avoid the induced fractures during cutting. Pure ethanol cooling liquid was used to cool the cutter instead of water to prevent water interaction with the shale before the treatment. To fit the hydrothermal reactor (shale treatment reactor), the shale specimen size must be smaller than 1 cm × 1 cm × 1 cm size. Several powder samples were also prepared before the treatment from four types of samples.

The sample surface was cleaned and then dried for 12 h in the oven at 70 °C to delete the humidity in the generated samples. Samples of collected shale blocks were broken into powder of 60–80 mesh, for XRD and low-pressure nitrogen adsorption measurements, before and after treatment with de-ionized water. The 60–80 mesh is the best size for the N_2_ carbon adsorption and XRD^31^.

### Shale treatment

**Figure 1:**
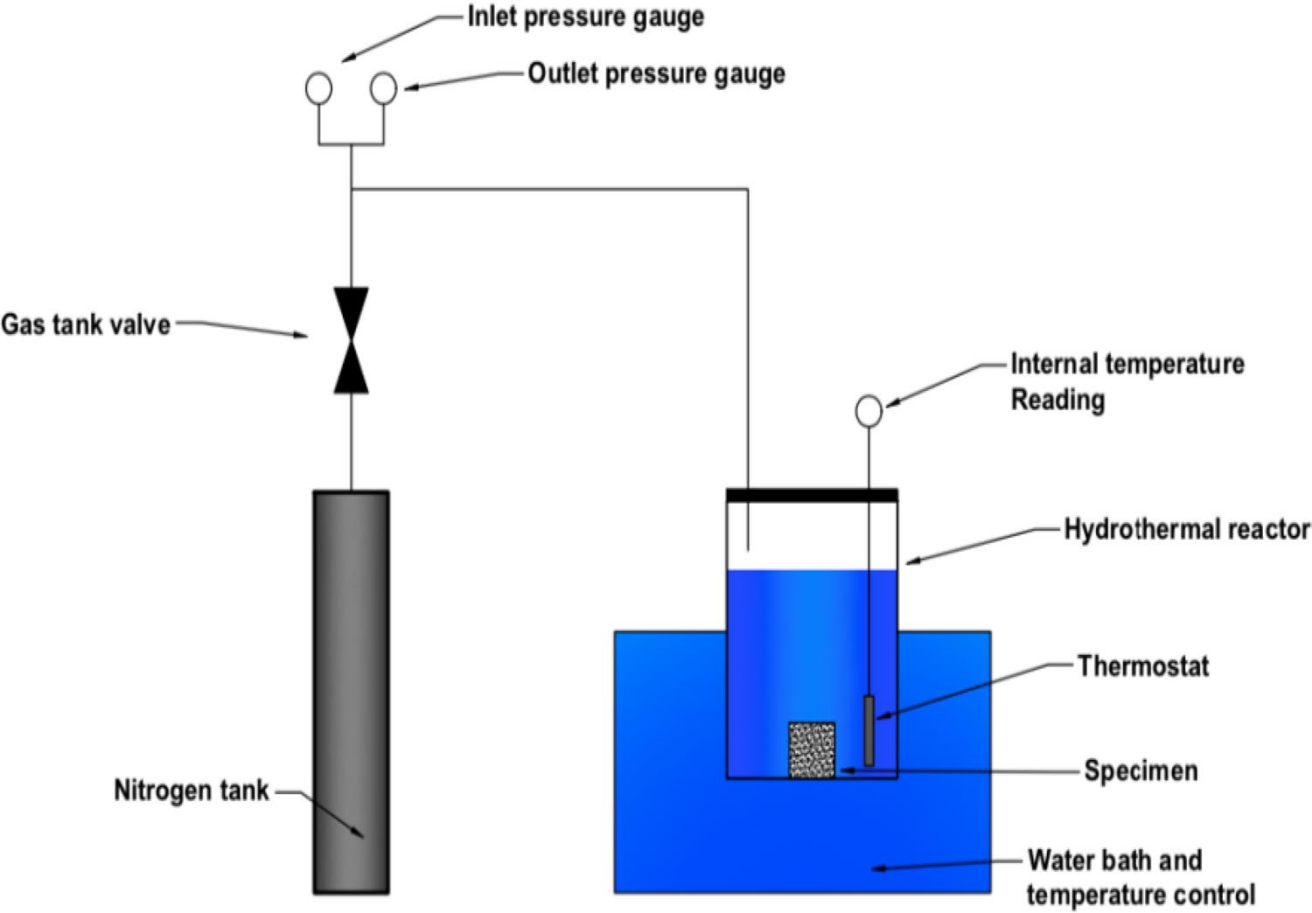
Shale treatment setup.

The shale treatment experiment was performed using a specially designed test setup, as shown in Fig. 1. The main component of the test setup consisted of a hydrothermal reactor. High pressure was supplied using a liquid nitrogen tank to this shale core treatment chamber, and the hydrothermal reactor was immersed in a constant temperature water bath. Before the treatment test, the setup connections were checked because each shale sample must be inside the chamber for three days to achieve the softening.

The reaction temperatures of the gas-bearing shales were subjected to 100 °C to simulate the temperature of the formation at depths of about 3 to 4 km with geothermal gradients of 20–25 °C/km^32^. Furthermore, the whole setup was checked for leaks before the tests to ensure it is fully sealed. Then, the treatment chamber was filled with DI (de-ionized) water, and the original cubic shale was fully immersed in this simulated fracturing fluid. Next, an 8 MPa pressure was applied to the hydrothermal reactor, and the reactor was kept inside a 100 ℃ constant temperature water bath for 72 h to simulate the initial field conditions^33–37^. Finally, the four types of shale samples were submerged in deionized water for the same periods to evaluate the clay-swelling and the alterations in the shale pore structure with time.

There were five main reasons for the use of deionized water, which are summarized below:

1. The concentration of electrolytes in water can reduce the potential swelling by reducing the diffuse double layer (DDL)^38^.
2. The high salt concentration in slick-water can affect the physical properties of clay by causing fine particles to bind together to aggregates or flocculate. This can reduce surface area and decrease free swell and swell pressure^39^.
3. The swell potential can decrease with cation exchange between water and clay and prevent the entry of water between the layers^40^. In addition, the DDL thickness decreases with salt concentrations leading to the collapse of the clay structure and reducing swelling potential^41^.
4. The high sodium ions can replace divalent ions such as calcium, which lower DDL. Both capillary hydration and surface–osmotic hydration have been studied and they can be combined to alter the quantity of water absorption into partly saturated shales. The fracture initiation is strongly dependent on confining stress and cracks originate parallel to weak features in rocks^42^. The shale used in this study are large block samples and there is no confining pressure was applied during softening simulations. Therefore, the swell potential decreases with an increased sodium concentration in the slick-water^43^.
5. The high temperature and pressure have a significant impact on shale-fluid interactions, which control shale treatment. The addition of chemicals will also increase mineral dissolution rate, intensified by high temperatures, leading to shale structure and integrity breakdown.

Hence, chemicals should be excluded when investigating the pure clay interactions in the shale formations.

### XRD measurements

The X-ray diffraction (XRD) data from four shale samples were used to determine the mineralogy. The mineralogy study is critical to explain the shale softening mechanism based on the clay content of shale. A more significant clay-water reaction is expected with higher clay contents in a shale formation^18,42^. Also, clay content varies over a wide range in different formations; hence the determination of clay content is essential to quantify the degree of shale softening. In addition, the carbonates may also contribute to shale softening. Hence, the mineralogy study included the quantification of both clay and carbonate contents using XRD tests. Thus, XRD was performed before and after the treatment tests for all shales.

The original and treated shale samples were cleaned, dried, and grounded to a size smaller than 120 μm to be used for mineralogical analysis. The same procedure was followed for treated shale. A copper source of 40 kV and 40 mA was used for the XRD, and the shale powders before and after treatment were subjected to diffraction angles (2θ) between 5° and 60° at a scan rate 1°/min^44^.

### BET measurements

The BET analysis aims to determine the absorption and desorption pattern of powder samples, in this case, shale before and after treatment, using the Autosorb machine (model type Autosrob-iQ manufactured by Quantachrome Instruments). All test samples were first dried in a vacuum for 12 h under a constant temperature of 150 °C before BET tests. Then, the BET measurements were made and the pore volume was determined using the Density Functional Theory (DFT) model^45–47^.

After 12 h of degassing and full isotherm gas sorption test, both sample powder before and after shale treatment were tested under the same conditions, including degassing pressure and temperature, loading condition, and liquid N_2_. Finally, the porous media-absorption and desorption graphs for four shale samples before and after treatment were obtained based on the raw data.

## Experimental results

### Density and minerology of shale

Before the treatment test, the density in g/cm^3^ of each shale was measured and found 2.29 for Opalinus, 2.53 for Haynesville, 2.58 for Longmaxi, and 2.43 for Eagle Ford.

**Figure 2:**
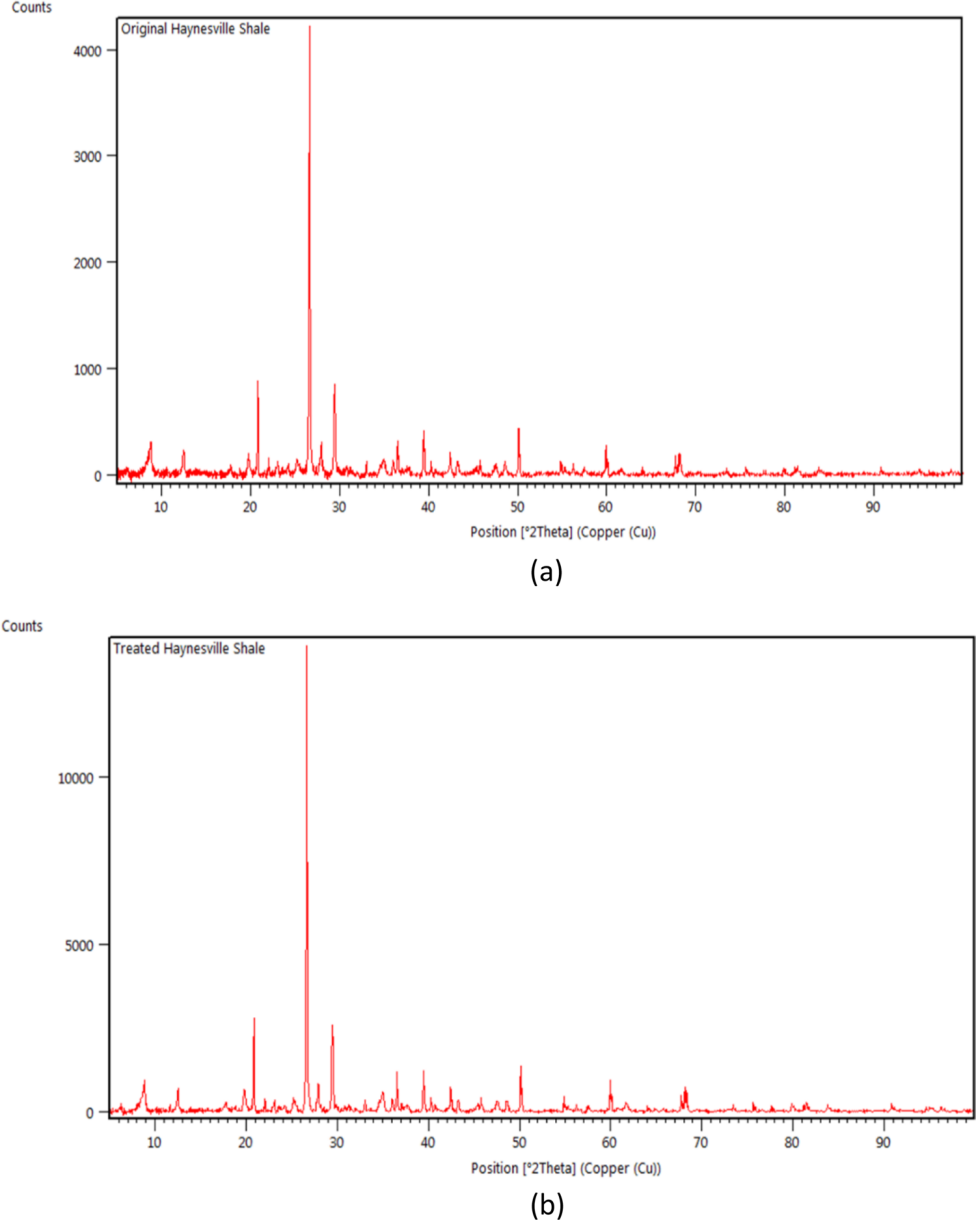
XRD patterns of Haynesville shale. (**a**) Original Haynesville Shale XRD. (**b**) Treated Haynesville Shale XRD.

The XRD results showed peak values for each crystalline material over the scanning angles. By matching the database of known crystalline patterns, one can quantify the mineralogy of a particular powder or solid using the Rietveld method^48–52^. Figure 2 shows the XRD results for Haynesville shale before and after softening treatment.

**Figure 3:**
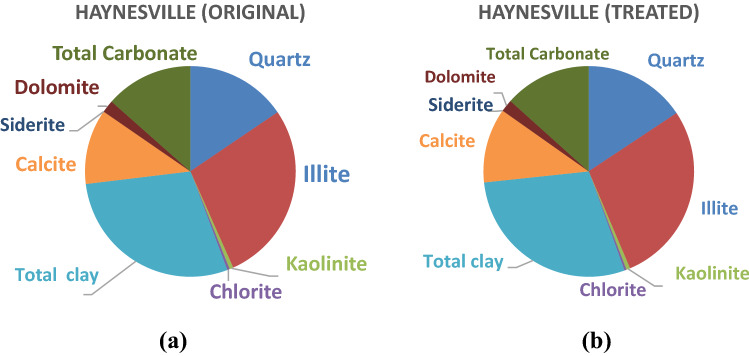
Mineralogy of Haynesville shale. (**a**) Original Haynesville Shale. (**b**) Treated Haynesville Shale.

Using high-score-plus, which is the advanced XRD data handling software, the quantification of mineralogy can be summarized into two pie charts for Haynesville shale, as shown in Fig. 3. Figure 3a shows the average original mineralogy of Hayesville shale: 24.1% Quartz, 43.2% Illite, 0.9% Kaolinite, 0.6% Chlorite, 2.5% Pyrite, 17.9% Calcite, and 3.0% Dolomite. Although there were minor differences between the measured and the reported, the measured results were comparable to those reported^53–56^. Also, the treated mineralogy of Haynesville is summarized in Table 1. As shown in Table 1, there was a 0.3% reduction of Illite (clay) and 0.4% reduction of carbonate reduction based on the Rietveld Quantification. The mineralogy of all four untreated shale are shown in Table 2.

**Table 1:** Mineralogical comparisons (Vol.%).

Haynesville shale	Quartz	IIlite	Kaolinite	Chlorite	Total clay	Calcite	Siderite	Dolomite	Total Carbonate	Others
Original	24.1	43.2	0.9	0.6	44.7	17.9	0	3	20.9	10.3
Treated	24.1	42.9	0.9	0.5	44.3	17.6	0	2.9	20.5	11.1

**Table 2:** Mineralogical composition of four shales (Vol.%)^53,54^.

Shale	Quartz	iIlite	Kaolinite	Chlorite	Total clay	Calcite	Siderite	Dolomite	Total carbonate
Opalinus	13.5	44.2	18.1	3.4	65.7	12.8	0.7	1.3	14.1
Haynesville	24.1	43.2	0.9	0.6	44.7	17.9	0	3.0	20.9
Longmaxi	36.3	35.6	0.8	0	36.4	2.0	0	10.3	12.3
Eagle ford	20.0	3.4	2.2	0	5.6	60	0	4.0	64

Please note that all cubic samples after softening treatment were all intact except for Opalinus shale with the highest clay content. This may be the reason that no hydraulic fracturing was performed in Opalinus shale formation.

### Absorption–desorption curves

**Figure 4:**
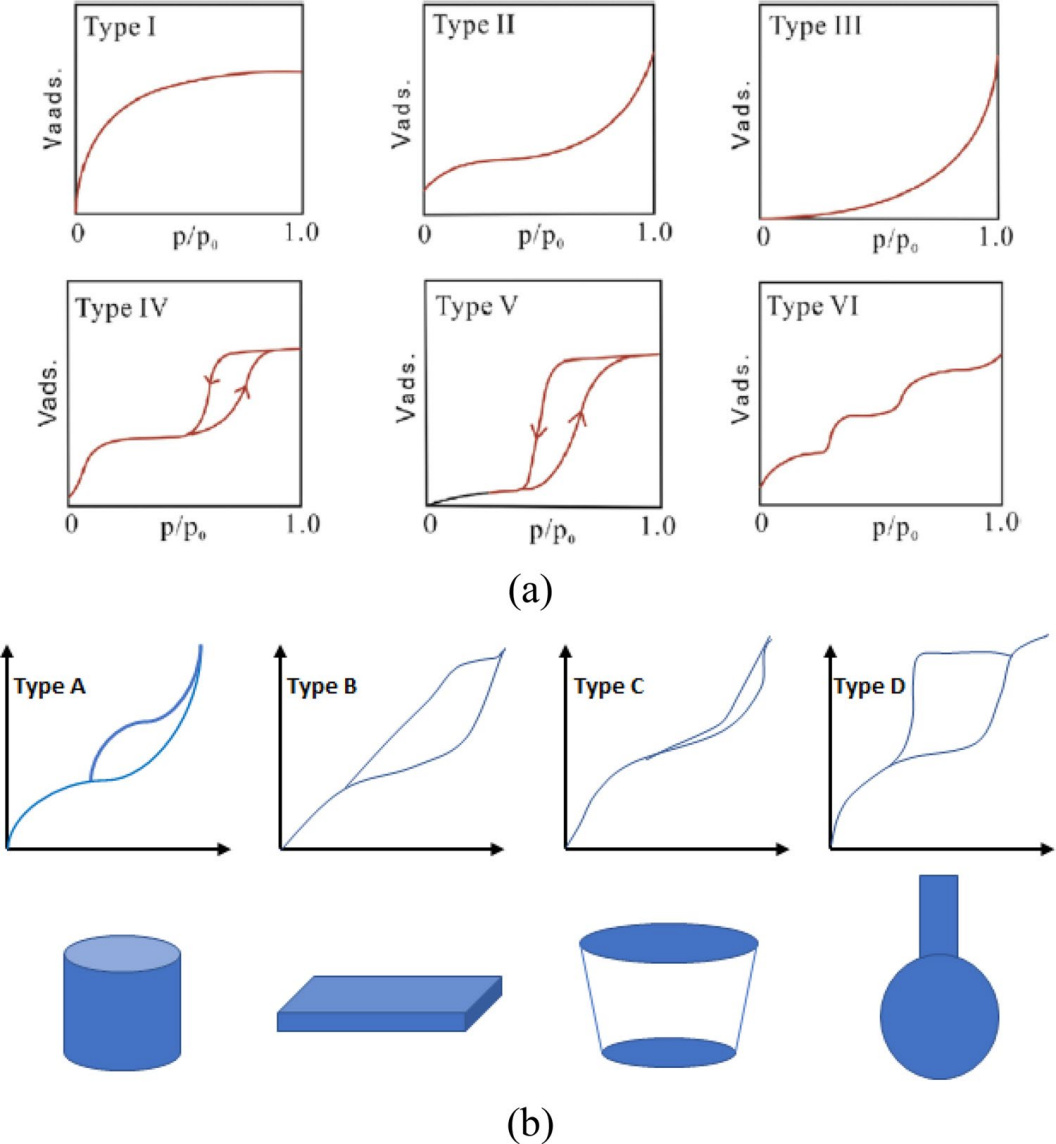
Adsorption isotherms types (**a**) and classification of hysteresis loops and their related pore shapes (**b**)^60,61^.

The shape and hysteresis of low-temperature N_2_ adsorption–desorption isotherms can effectively characterize the pore morphology of shale^57^. According to the classification of the International Union of Pure and Applied Chemistry (IUPAC), isotherms can be divided into six types (I to VI), and their hysteresis modes can be designated as Type A to Type D^58–60^ as shown in Fig. 4.

**Figure 5:**
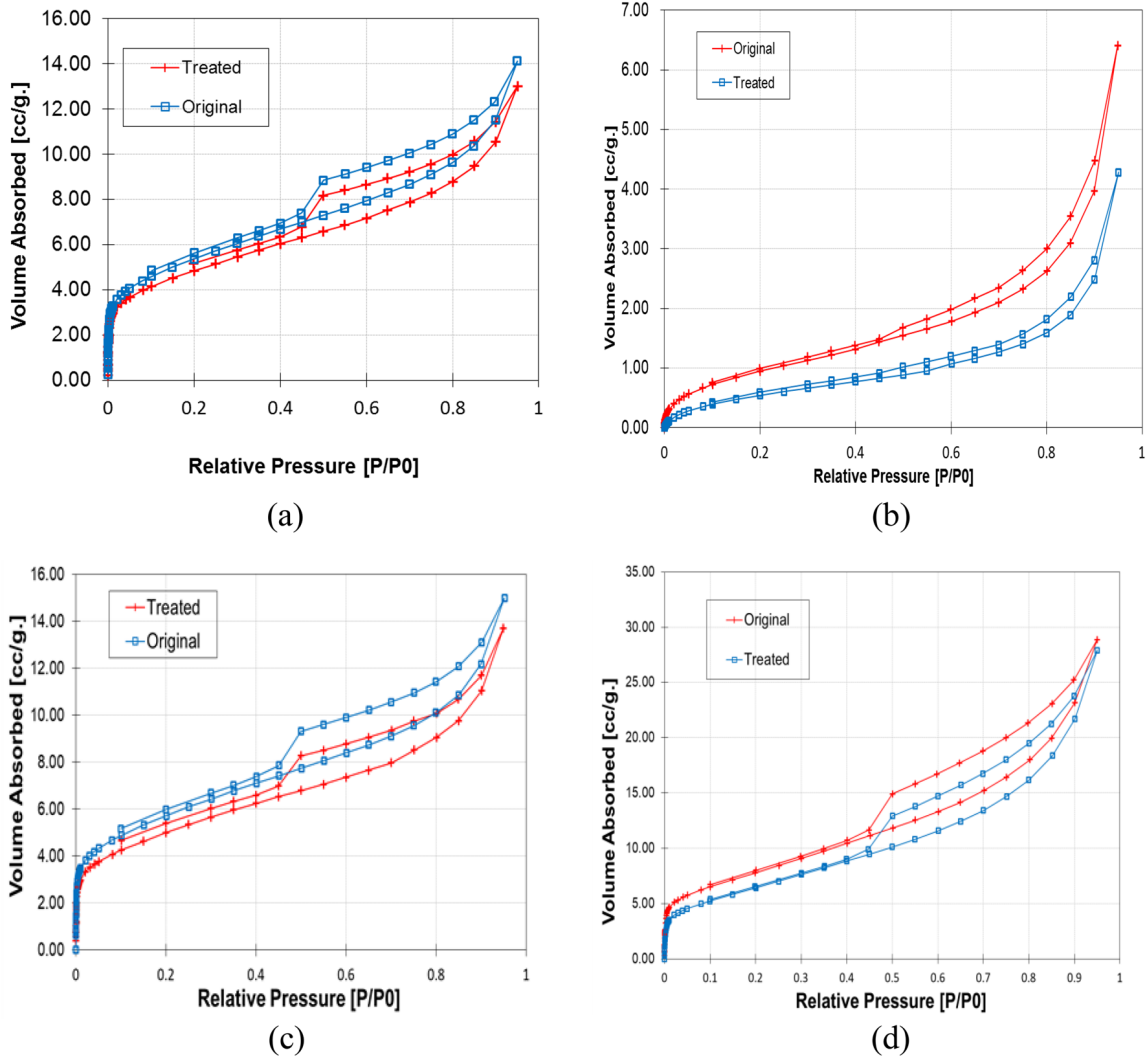
Isotherm Comparison for four shales tested. (**a**) Haynesville Shale. (**b**) Eagle Ford Shale. (**c**) Longmaxi Shale. (**d**) Opalinus Shale.

Figure 5 shows the measured N_2_ adsorption–desorption isotherms for four shales. According to the following isotherm graphs, the Opalinus shale N_2_ adsorption capacity dropped from 0.03668 to 0.03466 cubic centimeters per gram after treatment. Similarly, the maximum N_2_ adsorption capacity of Longmaxi, Eagle Ford, and Haynesville shales decreased from 0.0168 to 0.0148 cc/g, 0.04 to 0.032 cc/g, and 0.0177 to 0.0164 cc/g, respectively, indicating that the N_2_ adsorption capacity of all shale samples has decreased after treatment. The adsorption capacity is mainly related to the number of micropores and mesopores. Therefore, the change in N_2_ adsorption capacity of four shale samples is attributed to the change in the pore structure.

### Pore size distribution of original and treated shale

**Figure 6:**
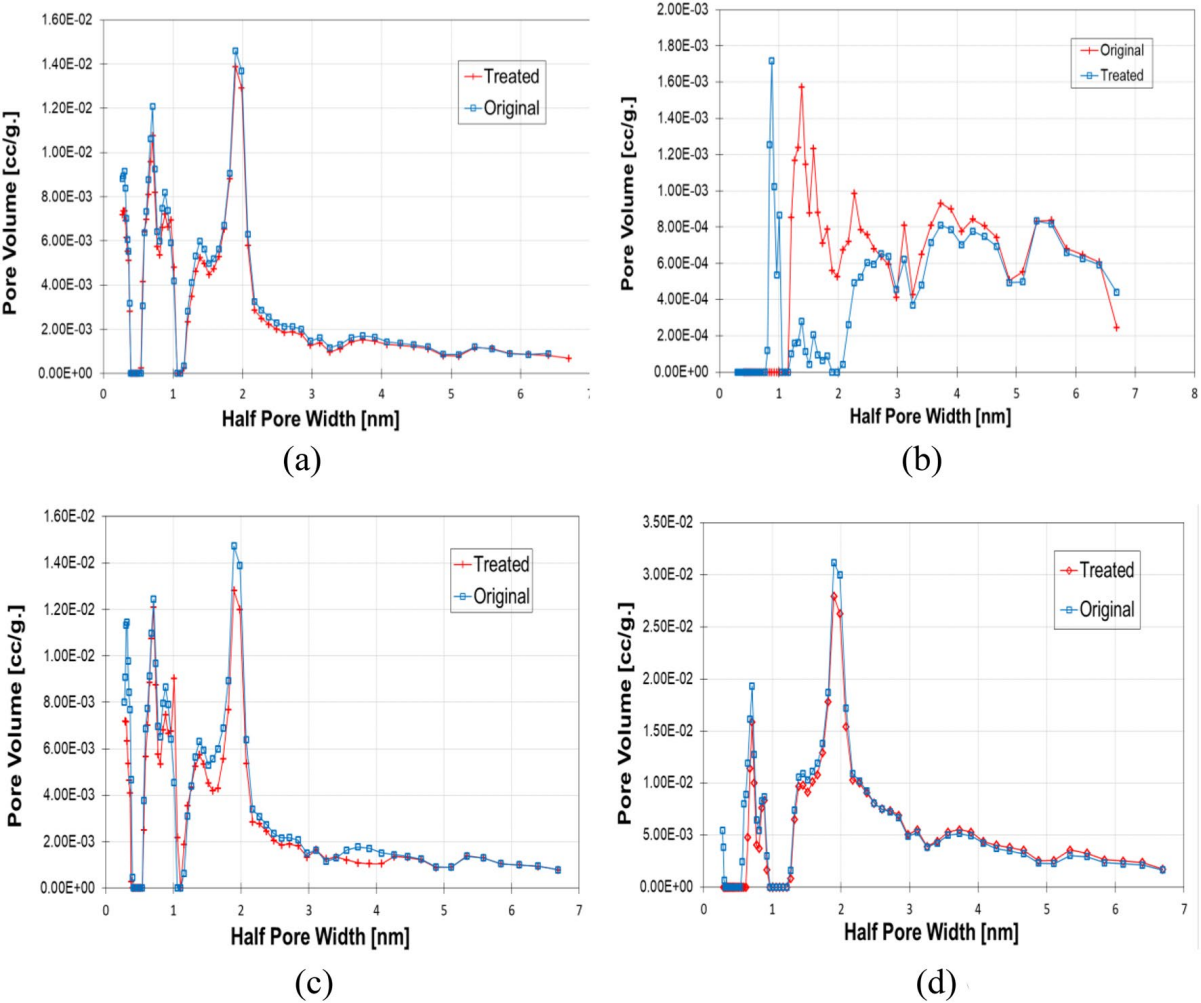
Pore size distribution comparison before and after treatment. (**a**) Haynesville Shale. (**b**) Eagle Ford Shale. (**c**) Longmaxi Shale. (**d**) Opalinus Shale.

The BET results can be used to obtain pore size distribution based on density functional theory (DFT)^59^, as shown in Fig. 6. The pore size distributions in Fig. 6 show subtle differences between the untreated and treated shale samples. However, the maximum amount of N_2_ adsorbed at the highest pressure changed.

Figure 5a shows the N_2_ adsorption–desorption isotherms Haynesville shale before and after treatment. According to the IUPAC classification, the N_2_ adsorption isotherms and hysteresis modes of all tested shale samples can be classified as Type IV isotherms^61^. At a lower relative pressure (P/P_0_ < 0.01), the amount of adsorbed gas in all tested shale samples is low, while at a higher relative pressure (P/P_0_ > 0.9), the amount of adsorbed gas increases sharply. This phenomenon indicates that micropores and mesopores with few large pores (larger than 50 nm) contribute to the total porosity of the sample. This hysteresis mainly presents sequential microporous and mesoporous materials arranged in a three-dimensional pore network based on the IUPAC classifications.

Figure 5b shows the isotherm comparison for Eagle Ford shale, the pattern of Type IV hysteresis. The Eagle Ford hysteresis loops are type C, demonstrating that in the Eagle Ford shales, slit-formed pores are the primary pores. Previous studies have shown the relation of plate-like pores to clay minerals^58,59^. The different compositions of clay minerals can cause variations in hysteresis loops types of Types IV and IV^62^. The type D hysteresis loops of the remaining three shale samples indicate that the pore shape of these shale samples may be bottle-shaped pores (with narrow necks and large pore bodies)^63^, as shown in Fig. 5b.

The pore size distribution can be used to quantify the changes in the pore structure of shale samples. The pore size distributions of the four shales indicate that the shale pore structure is multimodal, where at least two significant peaks can be found in all pore sizes. The pore volumes of four shales were reduced from 1 to 5 nm pore sizes. However, after treatment, the Eagle Ford shale pore volumes have significantly reduced when compared with the untreated samples, indicating a substantial change in number of micropores during treatment. Figure 6 shows that the peaks for the four shales are around 1 nm and 7 nm based on half pore width. Thus, the pore size distribution of the original four shales was altered after softening treatment.

Figure 6b shows a significant change in peak volumes for Eagle Ford shale from 2 to 6 nm. In this case, the large amount of inner Illite/Smectite (I/S) layer may contribute to a substantial change in pore sizes with clay swelling. Also, it was observed that after treatment from Fig. 6a,c,d, the pore volumes of Haynesville, Longmaxi, and Opalinus shale have slightly reduced from pore sizes 0.7 nm to 2.1 nm.

After integration, the cumulative volume of the four shales was calculated and is reported in Table 3. Before each BET test, the dry weight of powder samples was measured using a high precision scale. Hence, the porosity can be calculated by knowing the density of each shale. The volumetric density of each original and treated shale was obtained from BET analysis, and powder mass was used to calculate porosity.

**Table 3:** Variation in porosity between original and treated shale.

Sample	Volume/mass (original) (cc/g)	Volume/mass (treated) (cc/g)	Porosity (original) (%)	Porosity (treated) (%)
Longmaxi	0.0177	0.0164	1.58	1.06
Eagle Ford	0.0168	0.0148	4.40	3.80
Haynesville	0.0401	0.0322	2.94	1.84
Opalinus	0.0368	0.0346	7.80	3.80

The distributions of the pore size are shown in Fig. 6. The pore size distributions of Haynesville, Longmaxi, and Opalinus shales indicate that the shale pore structure is multimodal. It is shown that these three shale peaks are around 2 nm size, while the Eagle Ford shale peaks were around 1 nm size. It was also observed that the peak of pore size distribution of Eagle Ford shale shifted to 2 nm after treatment indicating the pore structure was altered due to clay-water interaction. While Fig. 6a,c,d show no evidence of shifting of peaks, and the I/S layer would be the primary reason for such peak changes. However, after the water-softening reaction, pore volume of all PSD of peaks decreased when compared to original samples.

**Figure 7:**
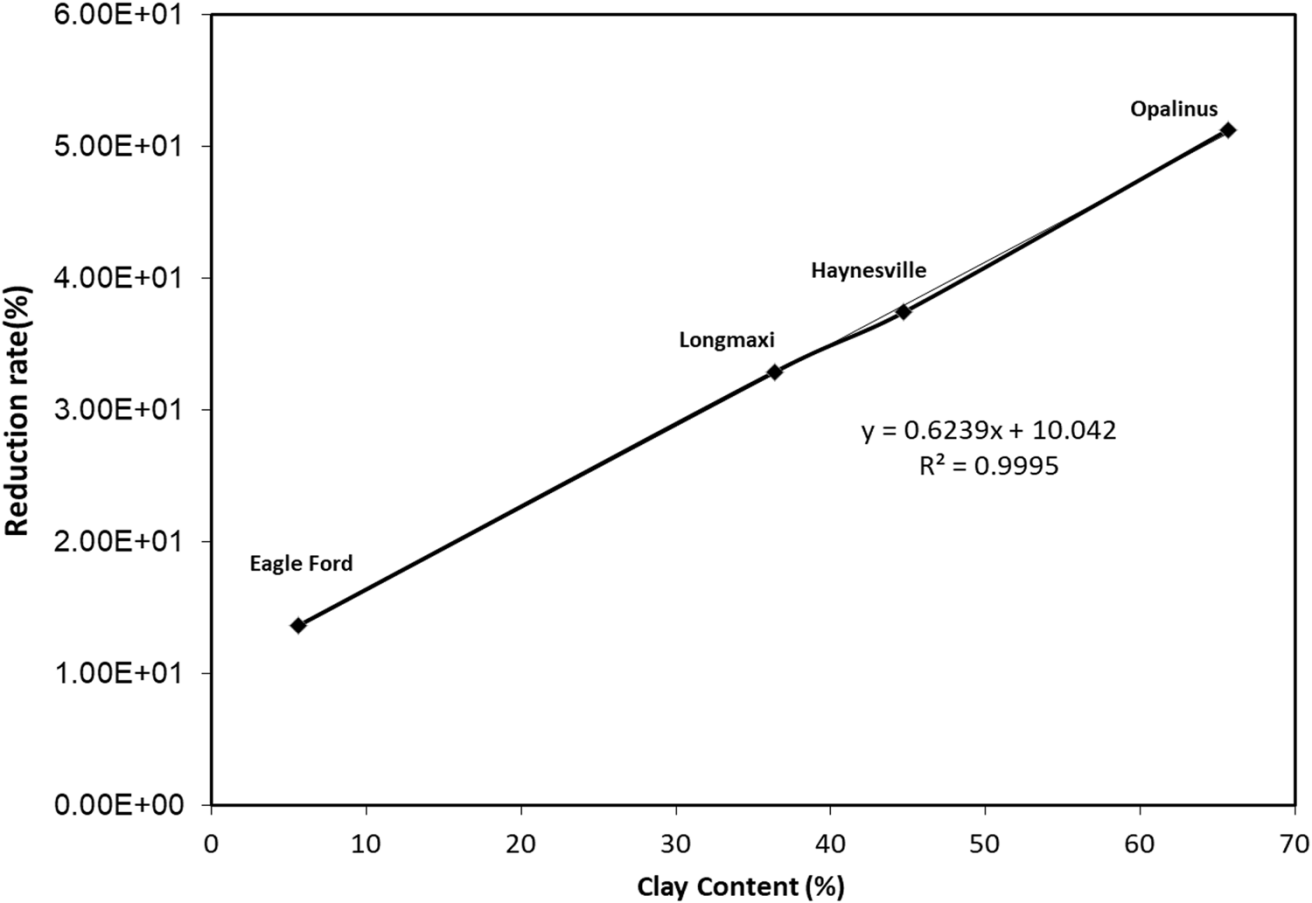
Correlation between clay content and porosity loss.

The clay content of tested shale varies from 65.7% (Opalinus shale) to 5.6% (Eagle Ford). Therefore, the reduction in porosity comparing original and treated shale of four was calculated and reported as the loss in shale porosity. The loss in porosity was plotted with the total clay content and shown in Fig. 7. A linear correlation between clay content of four shales and loss in porosity after softening was obtained and was reported as Y = 0.6239x + 10.042, R^2^ = 0.9995.

### Results and discussions

After shale softening or treatment tests on four different shales, there were several observations that described the clay content and porosity of shales. The change in isothermal diagrams of initial and treatment can be used to describe the pore structure of shale. The PSD analysis of all four shales before and after treatment, showed that changes in peaks can be used to calculate the porosity changes. Longmaxi, Haynesville, and Opalinus shales have the same isotherm pattern Type IV, and Eagle Ford shale may exhibit Type III. The Eagle Ford shale contains many I/S layers, contributing to lower swelling with 0.6% reduction of porosity.

A high clay content is generally considered to be detrimental to the reservoir during fracturing because it can reduce or block pore throats after hydraulic fracturing due to the retained injection fluid. During diagenesis, sediment porosity is substantially reduced for sediments with high clay contents but forms micro and nano pores and prevents cementation of Quartz covered with chlorite. Therefore, it is natural to associate the reduction in porosity of gas-bearing shale with high clay contents after hydraulic fracturing^64^. When clay minerals become unstable and react with injection fluids to transform into more stable minerals, these will precipitate on the surface of the matrix particles and expand the rock skeleton^65^. These stable clay minerals can block pore passages, reduce the connectivity between the pores, and increase the flow resistance^66^. Hence water-based fracturing fluids can significantly reduce the porosity and permeability of the reservoir through the swelling of clay minerals. As demonstrated in this research, the pore throats of high clay content shale gradually swell or block under the influence of high temperature and pressure, reducing the shale gas production with time^67^. This transformation of hard rock into mudstone or soft shale is usually called shale softening.

## Pore network model to simulate permeability based on pore structure

Reservoir rocks with natural gas are frequently anisotropic due to geological deposition or layering patterns. Because shale is highly anisotropic with bedding, permeability in the vertical direction is frequently lower than permeability in the horizontal direction^68^. This anisotropy was incorporated into the pore network used in this research (based on our previous publication^69^ and summarized in the enclosed Supplementary Materials). In this previous publication, anisotropy ratio incorporation was introduced, details of construction methodology was presented and model was validated by comparing model predictions with experimental results^69^.

The shale softening effect will have an enormous impact on the permeability and hence the shale gas production. Once the pore structure of treated or untreated shale is known, the pore network model can be used to predict the shale permeability to quantify the impact of shale softening. The equivalent pore network model is a simplified calculation model for porous media. Its structure is relatively simple, significantly reduce the complexity and calculating the seepage in porous media^69–72^. Therefore, this pore network model was used to predict shale permeability before, during fracking and after treatment.

**Figure 8:**
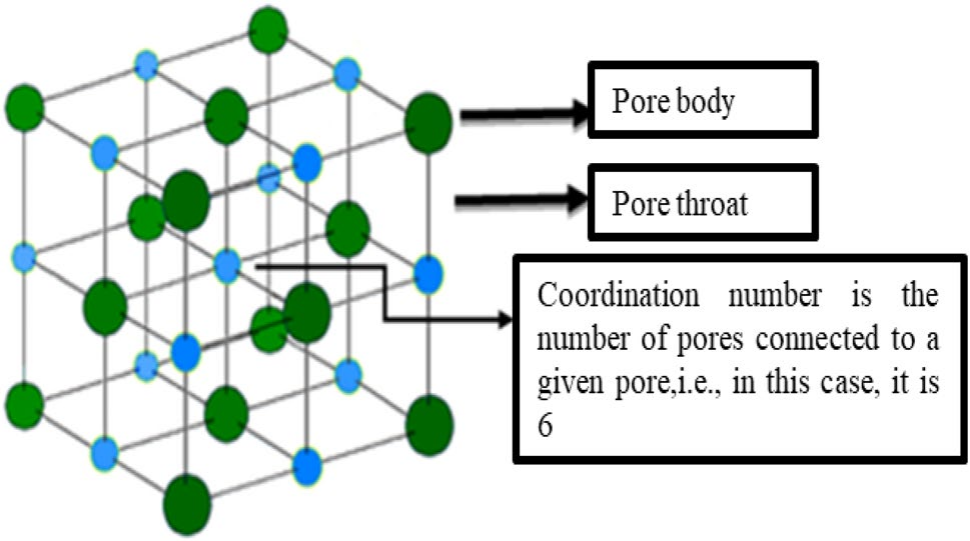
Schematic of each component.

As seen in Fig. 8, the pores or pore bodies in this model are spheres, and the pore mouths or pore throats are cylinders. Pore throats are linked to pore bodies. A pore can link up to a maximum of 26 pore throats. The coordination number is the number of pore throats associated with a specific pore. The average coordination number for the model is the average coordination number for all pores. The unit length is the distance between the sphere centers of two neighboring pores. The resultant model may readily replicate porous media with varying properties by changing the cell lengths, pore radius, pore throat radius, and the coordination number.

The following eight essential model parameters are needed to create the anisotropic pore network model:

1. Pore radius (Rp) and its distribution: The pore body radius represent the size of pore bodies in a geological medium, which are the large pores.
2. Pore throat radius (Rth) and its distribution: The throat radius represent the size of pore throats that facilitate flow of fluids. Because any fluid movement between the pores must pass via the pore throats, the pore throat size directly governs the seepage properties of the overall geological medium.
3. Coordination number (ϐ) and its distribution: A single pore body can be linked to numerous adjacent pore bodies in a geological medium. With high permeability, such as the sand, the coordination number is large. On the other hand, the pores coordination number is quite modest or low for low-permeability geological media such as shales.
4. Porosity (n): The proportion of void in geological media is represent the pores. All pores and throats, including dead pores and associated pore throats, are included in the porosity.
5. Characteristic length (L): The length of the mesh or lattice, which is the distance between neighboring pore bodies, is represented by the letter L in the pore network model.
6. Swelling ratio(s): S is defined as r_th(swelled)_/r_th(original)_. S is a concept introduced to the pore network model as an indicator to show different stages of softening in the shale matrix.
7. Swell layer (E): E is another concept introduced to the pore network model to account for shale softening. This factor also serves as an indicator that controls the swelling stages. The swell layer would be the first layer in contact or exposed to hydraulic fracturing fluid. Then it would propagate inwards.
8. Anisotropy parameter (ax, ay, az): The hydraulic properties of geo-materials in different directions differ due to distinct depositional patters resulting in anisotropic permeability. This hydraulic anisotropy is captured using the anisotropic parameter.

The volume filled by the pore body in a single pore unit is:

1 $${V}_{\mathrm{p}}=\frac{4}{3}\times \pi \times {R}_{\mathrm{p}}^{3}$$

The pores throat is assumed to be a cylindrical, which determines its volume by its length. Therefore, the length of the throat should be the distance between the centers of the pores, and the volume of the pores should be:

2 $${V}_{\mathrm{th}}=\frac{\upzeta }{2}\times \pi \times {r}_{\mathrm{th}}^{2}\times (L-2{R}_{\mathrm{p}})$$

With ζ, the coordination number, Rth the radius of the throat, and L the size of the unit, the porosity can be shown as:

3 $$n=\frac{{V}_{\mathrm{p}}+{V}_{\mathrm{th}}}{{L}^{3}}=(\frac{4\pi {R}_{\mathrm{p}}^{3}}{3}+\pi {R}_{\mathrm{th}}^{2}\times \left(\frac{L}{2}-{R}_{\mathrm{p}}\right)\times\upzeta )/{L}^{3}$$

Equation (3) connects porosity, pore radius, throat radius, pore coordination number, and unit length. Hence the five parameters needed for the construction of a basic PNM are the pore size, Rp, throat size, Rth, pore coordination number, ζ, the size of the lattice, L and porosity, n. Equation (4) can be employed to account for the anisotropy of connections^69^.

4 $$\frac{p\left(\alpha ,\beta ,\gamma \right)}{p\left({\zeta }_{i},{\zeta }_{j}\right)}=\frac{{a}_{x}{cos}^{2}\alpha +{a}_{y}{cos}^{2}\beta +{a}_{z}{cos}^{2}\gamma }{\overline{a} }$$

where α, β, and γ and are the angles formed by the connecting to the directions along x, y, and z-axes, respectively. The link between pore connection probability inside a lattice and their spatial angles to the x, y, and z directions is included in this equation^69^.

The model construction can be divided into the following steps:

- Generation of pore bodies and pore connections.
- Use the double-labeling method to eliminate all isolated pores in the equivalent pore network model.
- After the original network was established, each of the parameters was stored. Now assign the newly introduced swelling factors (swelling layers and swelling ratio) to the original constructed model.
- The swelled pore throats will result in the reduction of porosity as well. As the swelling continues, the reduction of porosity would continue. Each time step, the swelled porosity would be calculated.
- After the calculation of the porosity, the swelled pore network would repeat Step (1) to Step (4) until the new network is entirely constructed.

## Permeability of soften shale based on anisotropic pore network model

**Figure 9:**
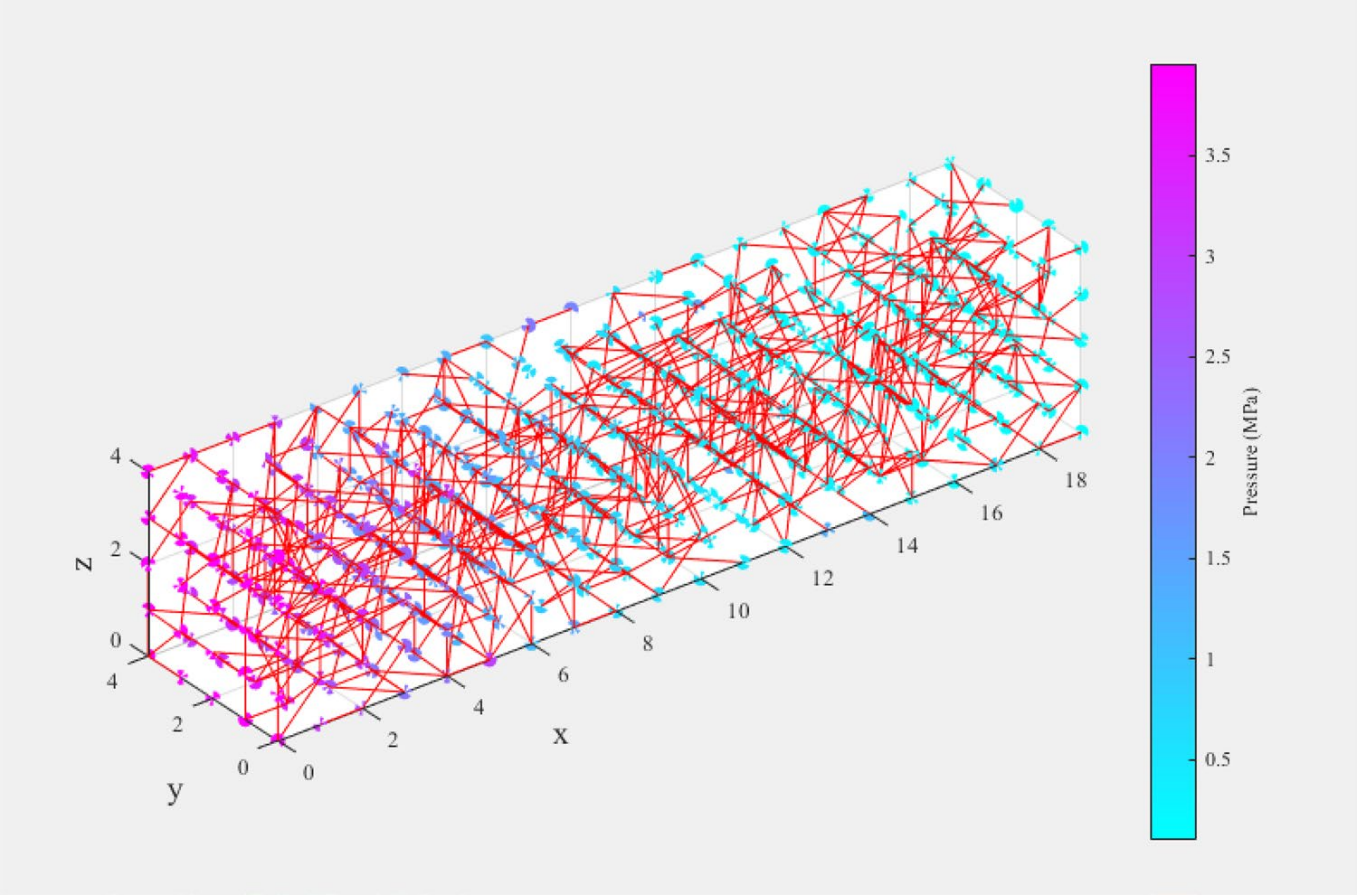
Illustration of a swelling pore network (Constructed using Matlab and C++).

Using this pore network and incorporation the change in porosity due to shale softening will provide insights into the change in shale permeability and help understand the damage the shale formation due to fracking. The permeability result will be compared based on the spatial structures and porosity of the original and treated shale. Figure 9 shows one quarter of the 20*20*20 network used for the simulation to illustrate the pore bodies connected to pore throats where pore bodies are spheres, and the pore throats are cylinders, and the pore bodies are connected by pore throats.

Based on several publications describing Longmaxi^69,71,73^, the PSD and the initial shale of porosity of 1.58%, the PNM for Longmaxi shale was reconstructed. Then reduced porosity was achieved by gradually reducing sizes of pore bodies and pore throats with a swell ratio of 0.71, as shown in Table 4.

**Table 4:** Original Longmaxi shale PNW parameters^70,71,73^.

Porosity	Model size	Mean pore body diameter (nm)	Mean pore throat diameter (nm)	Coordination number	Swell ratio	Anisotropic ratio (*a*_x_:*a*_y_;*a*_z_)
Original (1.58%)	20 × 20 × 20	4.3	0.66	4	0	22: 22: 1
Treated (1.06%)	20 × 20 × 20	3.1	0.46	4	0.71	22: 22: 1

Several assumptions were made for the simulation as follows:

- Assumed that swelling initializes from the first layer of the grid in contact with hydraulic fracturing fluid.
- Due to the clay-water reaction, the pore body and the pore throat swell, but only the throat contributes to the matrix permeability (see the Supplementary File for details).
- As the softening gradually progress, the first layer swells first and then continue to the next layer until the entire model is swelled.

**Figure 10:**
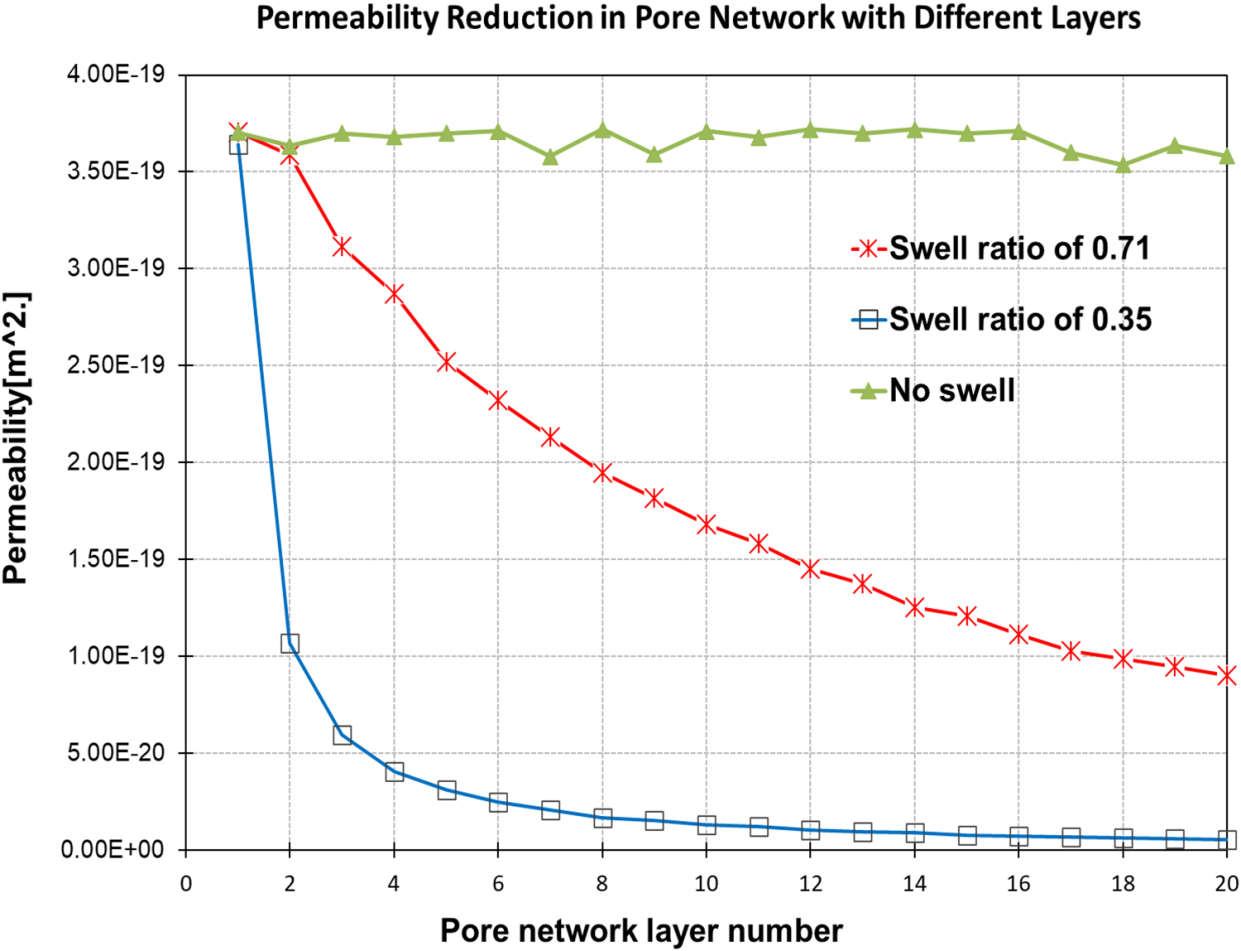
Impact of different swelling ratios on permeability of shale matrix.

To demonstrate the swelling, Fig. 10 shows an increase in swelling propagation through layers from no swelling to a swelling ratio of 0.35 and 0.71 for the layers in contact with hydraulic fracturing liquid, indicating swelling with the progress of fracturing. The outlet was fixed at 0.1 MPa and the inlet pressure was 0.2 MPa.

**Figure 11:**
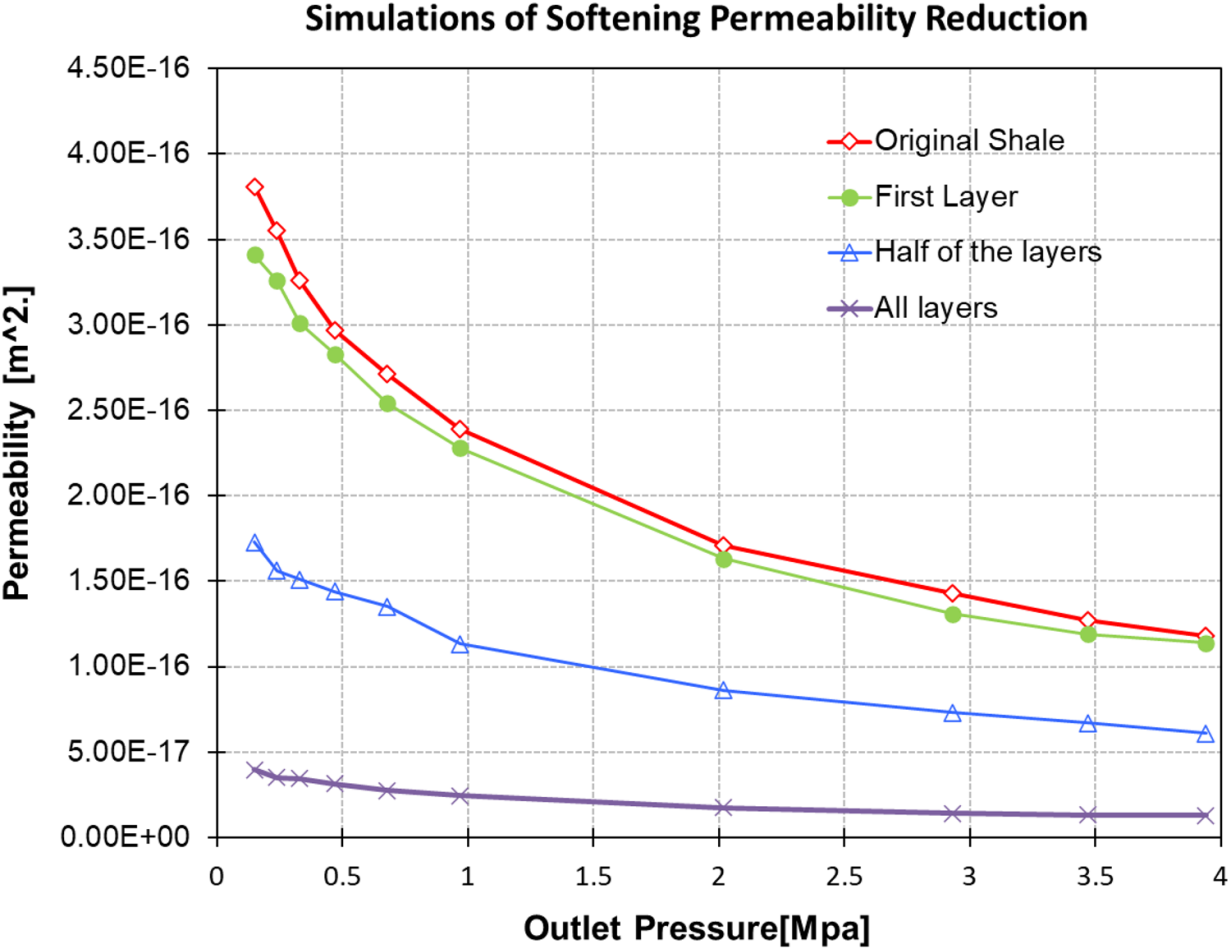
Permeability for different conditions of shale.

Four different simulations were performed to demonstrate the stages of shale softening, which are situation 1(no swelling, original shale), situation 2 (Layer one), situation 3 (half of the layers), and situation 4 (Treated shale), as shown in Fig. 11. These stages represent hydraulic fracturing progress in which water seepage from the first layers to the entire grid. Swell ratio and layer number were assigned to understand the alteration of shale matrix permeability during fracturing for different stages.

For this simulation the outlet pressure was fixed at 0.1 MPa, and the inlet pressure gradually increased from 0.1 to 4 MPa. The permeability decreased dramatically after half swelling to 42% of the initial permeability. For Longmaxi shale, even though the porosity reduced by only 0.5% of the original shale, the permeability reduced to 14% of the original or untreated shale.

The permeability of the original shale decreased dramatically as the swell ratio increased, concluding that softening of shale is the main reason for the loss of permeability of the shale matrix.

It is also reasonable to conclude that with moisture diffusion into the shale matrix, pore sizes in the whole lattice decrease, and hence the matrix permeability. In the extreme case, with swelling of all layers, the permeability of matrix would be substantially smaller than the original permeability.

The above change in permeability due to shale interaction with the fracking fluid will be incorporated into another publication to quantify and predict the impact of shale softening on the commercial production of shale gas^74^.

## Summary and conclusions

Four shale samples with different mineral compositions obtained from formations around the world were used to study the influence of initial shale mineralogy on pore porosity evolution during hydraulic fracturing. Using XRD, low-temperature N_2_ adsorption, and porosity measurements, the changes in mineral composition and pore structure characteristics of shale samples were measured. The conclusions of this study are summarized as follows:

1. In the clay-rich Opalinus shale (clay content of 65.7%), the clay softened after being immersed in DI water at high temperature and pressure. The expansion of IIlite in Opalinus shale caused swelling of nano pores and led to a significant decrease in total pore volume (shale porosity decreased from 7.8 to 3.8%).
2. In the clay-poor Longmaxi shale with a clay content of 1.58%, the clay is cemented with other non-water reaction minerals. The clay cementation may occur at a slow rate and the standard softening experiment cannot detect such cementation. However, the cementation of clay may clog pore throats. As a result, the porosity of the shale reduced from 1.58 to 1.06%.
3. The change in shale porosity with different mineral compositions during hydraulic fracturing may be related to the clay content of shale. Shales with low clay content have a low softening capacity, resulting in lower porosity loss after treatment. However, clay-rich shales have a high softening capacity. The test results showed that during hydraulic fracturing, the original mineral composition of the shale may impact the pore structure and hence shale gas recovery. Therefore, the fracturing fluid should be tested for compatibility.
4. The test results confirmed that shale softening has a negative impact on both the permeability of shale and gas production. Also, knowledge of structural parameters of shale such as pore size distribution, porosity and mineralogy can help to quantify shale softening. The testing or simulation of shale softening provides a scientific methodology to predict the changes of permeability.
5. A linear relationship between clay content and reduction in porosity indicated that clay-rich shale would have the worst damage due to hydraulic fracturing with water based fracturing fluids. Highest softening was observed for clay-rich shale: the Opalinus shale.
6. Permeability decreased with the propagation of swelling. The reduction of porosity will be the main reason for the reduction in permeability. Even though the clay swelling-related porosity only decreases by 0.52%, the permeability of treated shale was only 14% of that of untreated shale.

## Supplementary Information

Supplementary Information.
