# Supplementary material for: Impact of de-ionized water on changes in porosity and permeability of shales mineralogy due to clay-swelling
Source: Sci Rep. 2021 Oct 8;11:20049. doi: 10.1038/s41598-021-99523-2 (PMC8501039; doi:10.1038/s41598-021-99523-2)
Supplement: Supplementary file 1 — Supplementary Information. [file 41598_2021_99523_MOESM1_ESM.docx]

**Supplementary document**

An equivalent pore network is a simplified pore structure of the geo-media. It simplifies the complex flow paths (pore bodies, pore throats, etc.) in the geo-media into regular geometric shapes, such as spheres and cylinders, as shown in Fig 1.


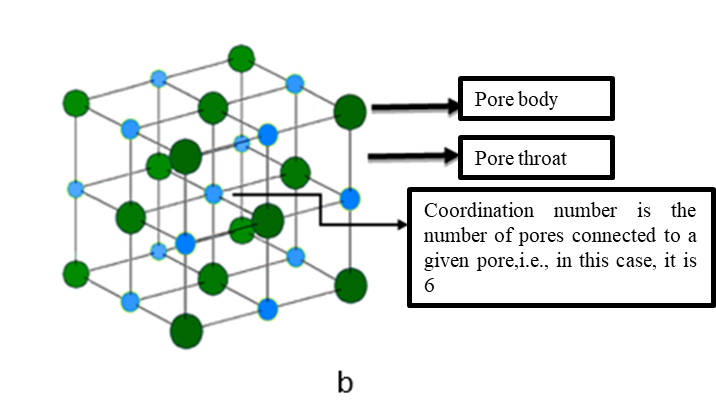


Figure 1. Schematic of equivalent pore network model

This methodology evolved from two-dimensional to three-dimensional model and now developed into equivalent pore network models suitable for simulation of various geo-media. The pore bodies in an equivalent pore network represent the larger sized cavities in the geo-media. The pores are connected by pore throats, smaller cavities, or nano-fractures for fluid migration. In an equivalent pore network, the size of pore radii varies. They are regularly arranged on grid points. The distances between the centers of adjacent pores is a constant value to simplify the calculation. The average number of surrounding pores varies depending on the geo-material. The average number of active connections to a pore body is called the coordination number. By adjusting the size of the pore radius, pore throat radius, and the pore coordination number, a pore network model can be developed to simulate the gas seepage within geo-media [1].

In the equivalent pore network model constructed in this study, the non-solid phase includes pores and pore throats, and its porosity is:

$n=\frac{Vp+V_{\mathrm{th}}}{L^{3}}=\frac{\frac{4\pi r_{p}^{3}}{3}+\pi r_{\mathrm{th}}^{2}\times\left( \frac{L}{2}-r_{p} \right)\times}{L^{3}}$ (1)

Where ζ is the coordination number, r_th_ is throat radius, and L is the unit size.

When the average coordination number in the equivalent pore network model is low, then there may be cases where the coordination number of some pores may be less than 2, and hence becomes dead-end pores (coordination number is 1) or isolated pores (coordination number is 0). There may also be dead-end pore groups and isolated pore groups (multiple pores have less than two connections to the main percolation channels). These pores are in the actual pore structure, but they do not contribute to the flow, so in the equivalent pore network model, they can be eliminated, thereby reducing computation time.

Actual geo-media are usually anisotropic, and different directions often exhibit different permeabilities. In the process of model building, an anisotropic parameter can be used in the equivalent pore network with different connection probabilities. Since the developed pore network model is three dimensional, a new concept was introduced to consider the anisotropic permeabilities for three different planes of shale matrix: anisotropic ratio. It is defined as the ratio of the number of pore connections in the three directions of x, y, and z as (a_x_, a_y_, a_z_); since the developed model is a regular lattice grid, the total number of connections for each plane is available after the construction also for the angel between connections to x, y and z axis, in this case (regular lattice cubic grid), angles between the space diagonal to each of the axis within a cubic would be 54.7° defined as α, β and γ. When the certain shale media of given anisotropy ratio (a_x_, a_y_, a_z_), the number of pore connections in the three directions of x, y, and z are first guaranteed. To calculate the probability in other directions (α, β and γ directions) other than x, y and z directions, the equation 2 can be used and connections to this lattice model are determined based on this connection probability.

$\frac{p\left( \alpha,\beta,\gamma\right)}{p\left( \zeta_{i},\zeta_{j} \right)}=\frac{a_{x}{cos}^{2}\alpha+a_{y}{cos}^{2}\beta+a_{z}{cos}^{2}\gamma}{\bar{a}}$ (2)

Considering one-dimensional seepage, when the fluid forms a stable flow field, the permeability of the entire equivalent pore network model can be calculated based on Darcy's as shown below:

$Q=\frac{kA}{\mu}\frac{dp}{dL}$ (3)

Where Q is the volume flow, k is the absolute permeability of the medium, A is the seepage area, μ is the viscosity of the fluid, L is the seepage distance, and dp / dL is the pressure gradient.

Considering the compressibility of gas, in the case of stable seepage, the volume flow of upstream and downstream may be different, but the mass flow must be the same, so the above formula must be expressed as mass flow, which is:

$Q_{m}=\frac{kA}{\mu}\frac{dp}{dL}$ (4)

Where ρ is the density of the fluid.

When the seepage fluid is a gas, the density of the gas gradually changes with the pressure.

$\frac{dp}{dL}=\frac{\mu}{\rho}\frac{Q_{m}}{kA}=\frac{\mu}{p}\frac{RT}{M}\frac{Q_{m}}{kA}$ (5)

After integration:

$p_{i}^{2}-p_{0}^{2}=\frac{\mu RT}{M}\frac{Q_{m}}{kA}L$ (6)

Organized into a form similar to liquid phase seepage to obtain:

$k=\frac{2\mu RT}{{(p}_{i}+p_{0})M}\frac{L}{A}\frac{Q_{m}}{p_{i}-p_{0}}=\frac{\mu}{\bar{\rho}}\frac{L}{A}\frac{Q}{p_{i}-p_{0}}$ (7)

Where $\bar{\rho}$ represents the average density of gas in the entire pore network. Regardless of the type of seepage fluid, the permeability can be calculated using the above formula after obtaining the mass flow of the equivalent pore network model.

When analyzing the relationship between the geometric parameters of the pore network, it is found that some geometric parameters are mutually dependent. At a known element length ratio l_x_: l_y_: l_z_, the porosity θ, and the unit length l_x_ can be calculated from each other. In this study, the pore radius r, coordination number ζ, unit length ratio l_x_: l_y_: l_z_, connection number ratio a_x_: a_y_: a_z_, pore throat radius r_th_, porosity θ total six parameters are used for model construction, and these six parameters are independent and do not affect each other. These model parameters can be extracted from the real pore structure of porous media from previous studies.

The shape of the pores and reconstruction of the pore network model can be verified using the image analysis, especially for Longmaxi shale. Also, the low-pressure absorption test can support the assumption of equivalent spherical and cylindrical pore shapes [2]. Therefore, to understand the characteristics of the pore structure and to facilitate prediction of gas flow, the pore structure in the shale matrix was simplified and represented as a cylindrical capillary [3, 4, 5]. Hence the shale matrix pores consist of a large number of multi-diameter cylindrical pores connecting to spherical pore bodies in series and parallel.

The size of each pore network was 20×20×20. The pore diameters and porosity values were obtained from the experimental data reported in Gao et al. [6, 7]. In that study, they conducted different tests, including CO_2_ absorption, N_2_ adsorption, and mercury intrusion, to obtain pore structures of four Longmaxi shales under laboratory conditions. Other parameters, including the pore throats diameters, coordination numbers, were based on our previous and current research on shale[8, 9, 10].

References:

1. Gao, S., Meegoda, J. N. & Hu, L. Two methods for pore network of porous media. *IJNAM* **36**, 1954-1970, doi: <https://doi.org/10.1002/nag.1134> (2012).
2. Tian, H., Zhang, S. C., Liu, S. B. & Zhang, H. Determination of organic-rich shale pore features by mercury injection and gas adsorption methods. *Acta Petrolei Sinica* **33**, 419-427 (2012).
3. Zhang, H., Zhong, Y., She, J. & Li, G. Characterization of shale matrix pore structure via experiment and model. *Arabian Journal of Geosciences* **11**, 1-9, doi:10.1007/s12517-018-3698-9 (2018).
4. Zhao, B., Shang, Y., Jin, L. & Jia, B. in *Spe/aapg/seg Unconventional Resources Technology Conference.*
5. Wang, C. *et al.* Organic and Inorganic Pore Structure Analysis in Shale Matrix With Superposition Method. (2014).

6. Gao, J., Xia, L., Li, Y. & Yu, Q. Percolation characteristics of Carboniferous shale gas in Eastern Qaidam Basin. *Earth Science Frontiers* **23**, 103-112 (2016).

7. Gao, J., Yu, Q. & Lu, X. Apparent Permeability and Gas Flow Behavior in Carboniferous Shale from the Qaidam Basin, China: An Experimental Study. *Transport Porous Med* **116**, 585-611, doi:10.1007/s11242-016-0791-y (2016).

8. Zhang, P., Hu, L., Meegoda, J. N. & Gao, S. Micro/Nano-pore Network Analysis of Gas Flow in Shale Matrix. *Sci Rep* **5**, 13501, doi:10.1038/srep13501 (2015).

9. Zhang, P., Hu, L. & Meegoda, J. N. Pore-Scale Simulation and Sensitivity Analysis of Apparent Gas Permeability in Shale Matrix. *Materials (Basel)* **10**, doi:10.3390/ma10020104 (2017).

10. Zhang, D., Zhang, X., Guo, H., Lin, D., Meegoda, J. N., and Hu, L., (2021), “An Anisotropic Pore-network Model to Estimate the Shale Gas Permeability,” Scientific Reports, https://doi.org/10.1038/s41598-021-86829-4
